# Effects of China’s urban basic health insurance on preventive care service utilization and health behaviors: Evidence from the China Health and Nutrition Survey

**DOI:** 10.1371/journal.pone.0209890

**Published:** 2018-12-31

**Authors:** Wanyue Dong, Jianmin Gao, Zhongliang Zhou, Ruhai Bai, Yue Wu, Min Su, Chi Shen, Xin Lan, Xiao Wang

**Affiliations:** 1 School of Public Health, Health Science Center, Xi’an Jiaotong University, Xi’an, Shaanxi, People’s Republic of China; 2 School of Public Policy and Administration, Xi’an Jiaotong University, Xi’an, Shaanxi, People’s Republic of China; 3 Global Health Institute, Health Science Center, Xi’an Jiaotong University, Xi’an, Shaanxi, People’s Republic of China; 4 International Business School Suzhou, Xi’an Jiaotong-Liverpool University, Suzhou, Jiangsu, People’s Republic of China; St Luke's International University, JAPAN

## Abstract

**Background:**

Lifestyle choices are important determinants of individual health. Few studies have investigated changes in health behaviors and preventive activities brought about by the 2007 implementation of Urban Resident Basic Health Insurance (URBMI) in China. This study, therefore, aimed to explore whether URBMI has reduced individuals’ incentives to adopt healthy behaviors and utilize preventive care services.

**Methods:**

Data were drawn from two waves of the China Health and Nutrition Survey. Respondents were categorized according to their insurance situation before and after the URBMI reform in 2006 and 2011. Propensity score matching and difference-in-differences methods were used to measure levels of preventive care and behavior changes over time. Estimations were also made based on gender, self-reported health, and income.

**Results:**

We found that URBMI implementation did not change residents’ utilization of preventive care services or their smoking habits, drinking habits, or other risky behaviors overall. However, the likelihood of sedentariness did increase by five percentage points. Females tended to be more sedentary while males were less likely to drink soft drinks. Residents with poor self-reported health exercised less while those who reported good health were more likely to be sedentary. Low- and middle-income residents were likely to be sedentary while middle-income people tended to smoke after becoming insured.

**Conclusion:**

Since URBMI implementation, some unhealthy behaviors like sedentariness have increased among those who were newly insured, and different subgroups have reacted differently. This suggests that the insurance design needs to be optimized and effective measures need to be adopted to help improve people’s lifestyle choices.

## Introduction

To improve the health of residents, China has launched a series of health reforms, including establishing three basic health insurance programs targeting different groups. Among these, the Urban Employee Basic Medical Insurance (UEBMI) and Urban Resident Basic Medical Insurance (URBMI) were implemented for urban residents in 1998 and 2007, respectively[1], while the New Rural Cooperative Medical Scheme (NRCMS) was implemented for rural residents in 2003[2].

A number of studies have investigated the effect of insurance in China on the use of medical services[3], and there is evidence that URBMI has improved self-reported health status to some extent and prompted greater utilization of medical services[4–6]. However, relatively few studies have investigated the effect of insurance on preventive care service utilization and health behaviors, especially in consideration of the differences between types of insurance and subsamples. This gap exists despite the fact that studies investigating the effects of NRCMS found that insured rural residents tended to smoke, drink, and engage in other risky behaviors more so than uninsured people in rural China [7, 8].

What exactly is the effect of insurance on the utilization of preventive care services and the adoption of healthy lifestyles? The evidence from developed countries is inconclusive. The well-known Rand Health Insurance Experiment found that health insurance had no significant effect on weight, physical activity, smoking, or alcohol consumption [9]. Another analysis based on a nationally representative sample found that insurance was not associated with significant changes in health behaviors but was associated with increases in preventive care [10]. Moreover, research conducted in the US has found that insurance strongly encouraged heavy smoking and sedentariness [11]. Similarly, research on Medicare confirmed a reduction in physical activity just before receiving Medicare [12]. A study based on the Portuguese Health Survey also found that holding voluntary private health insurance decreased the likelihood of engaging in sports [13]. Evidence from the UK, however, indicated that private health insurance did not reduce preventive activities such as exercise or regular checkups [14].

Regarding body mass index (BMI), one study, using data from the Behavioral Risk Factor Surveillance System, found that health insurance coverage reform in Massachusetts was associated with a reduction in BMI [15]. Other studies, however, have found that Medicaid expansion had a positive correlation with BMI among nonelderly adults or diabetics [16, 17].

Research focused on women has found that welfare reforms in the US increased drinking among single mothers while smoking and weight gain increased among pregnant women with the expansion of Medicaid [18, 19]. A study of elderly American males found that those who had recently obtained health insurance reduced preventive behaviors and increased unhealthy behaviors [20]. Among younger people, meanwhile, one study found that health insurance decreased heavy alcohol consumption among young adults while another found increased use of preventive care, such as checkups, for children [21, 22]. One cross-sectional study in Colombia found that insurance did not reduce preventive care among patients with diabetes [23].

Similar studies are fairly rare in the context of developing countries. One study found that Universal Health Coverage in Thailand had a positive effect on annual checkups while another found that the Seguro Popular Experiment in Mexico negatively affected the use of preventive care services [24, 25]. A study in Ghana investigated the relationship between insurance and malaria prevention and found that insured households were less likely to sleep under insecticide-treated bed nets [26].

The World Health Organization (WHO) has declared that lifestyle choices are important determinants of individual health [27]. However, with the expansion of basic health insurance in China, there is insufficient evidence to clarify the effect on urban residents’ health behaviors. The present study, therefore, investigated through longitudinal comparison whether health insurance reduced incentives to pursue preventive activities and healthy behaviors among urban residents in China.

## Methods

### Data

Data were drawn from the China Health and Nutrition Survey (CHNS), which is an ongoing international collaborative project between the University of North Carolina and the Chinese Center for Disease Control and Prevention. The CHNS has had nine waves to date (1989, 1991, 1993, 1997, 2000, 2004, 2006, 2009, 2011), detailed descriptions of which can be found at the official website (http://www.cpc.unc.edu/projects/china).

The data used in this study came from urban residents who completed the adult questionnaires in the 2006 and 2011 waves, comprising 3,360 and 5,361 adults, respectively, accounting for approximately one-third of respondents. Those who did not complete both survey waves were excluded, and a sample of 2,020 respondents was thus obtained. After processing for errors and missing data, we obtained 1,934 respondents. Based on the research objective, the following were also excluded: 749 respondents who initially had no insurance but had other basic medical coverage in 2011, 518 who changed insurance schemes or became uninsured during the survey period, and 64 who had other supplementary insurance schemes in both waves. Thus, a total sample of 603 respondents was obtained. According to their insurance situation, 378 respondents who had UEBMI for the entire period 2006–2011 were classified as the control group, while 225 respondents who had no basic medical insurance in 2006 but participated in URBMI before 2011 were classified as the intervention group.

### Variables

#### Preventive care service utilization

All respondents were asked if they had received any kind of preventive care service in the last four weeks. Preventive care services can include check-ups, visual activity tests, blood tests, hypertension examinations, tumor examinations, and so forth. Respondents were categorized into two groups according to their answers (yes or no).

#### Health behaviors

Multiple indicators were selected to measure an individual’s health behaviors, including whether they currently smoked or drank alcohol or soft drinks. Physical activity was measured by a binary variable based on the respondent’s answer to the question of whether “he or she engages in activities such as Kong Fu, dancing, running, swimming, and playing ball games.” Sedentariness was classified according to the level of engagement in activities such as watching television, recreational computer use, online chatting, playing board games, or reading newspapers or magazines [7]. Meanwhile, a dummy variable indicating whether an individual’s BMI exceeded the WHO recommended threshold (BMI>25) was also created, based on the results of physical examinations conducted by doctors, nurses, or other health workers.

#### Other variables

Age, gender, marital status, educational level, work status, annual household income, number of people in the household, and region were selected to control for natural and social characteristics. Whether the participants had been diagnosed with a chronic disease and their self-reported health status were selected to control for health status. Answers to the survey questions on chronic diseases relied on doctors’ diagnoses of hypertension, diabetes, myocardial infarction, or stroke. A value of 0 was assigned if none of those diseases was reported while 1 was assigned if at least one was reported. The respondents’ self-reported health was grouped into two categories according to their answers (good or poor).

### Statistical analyses

#### Propensity score matching

To estimate the effects of insurance on preventive care service utilization and health behaviors, it was necessary to distinguish between an intervention group (D = 1) that had experienced insurance transition and a control group (D = 0) that had not. Although the intervention group had been exposed to URBMI, the comparison sample remained heterogeneous given that insurance uptake is nonrandom. To solve this problem, propensity score matching (PSM)—proposed by Rosenbaum and Rubin as the probability of receiving a particular intervention given the site of the correlate X_i_ [28]—was used to balance the characteristics of insurance participation between the two groups [29].

Following this strategy, a probit model was used to regress the intervention status on all baseline correlates and indicate the probability of being insured with the propensity score [30, 31]. Various PSM techniques have been adopted in the literature [32]. Following the approaches described above, we adopted a 5-nearest neighbor (NN) matching method by matching each intervened individual with five individuals in the control group who were close to him or her in the propensity score, as this indicated the best balancing properties among the correlated variables [33]. To assess the quality of the matching, correlate balance was tested using two-sample t-tests for both groups before and after the match. S2 Table shows the results of the correlate balance check. The correlates were balanced in both groups and became statistically indistinguishable after matching, as suggested by Rosenbaum and Rubin [28].

#### Methods of estimating intervention effects

The difference-in-differences (DID) method combined with PSM was used to estimate the effects of insurance on preventive activities and health behavior changes. Compared to the matching approach alone, the DID matching estimator relaxes the independence assumption between outcome and program participation, which means any bias caused by time-invariant unobserved systematic differences common to URBMI and UEBMI can be implicitly controlled [34].

For each outcome (Y_i_), the change in the intervention group and the change in the control group were compared during the study period to estimate the average treatment effect (ATT). The DID propensity score matching estimator was based on the following identifying assumption [35, 36]:

$${ATT}^{DID-PSM}=\frac{1}{N_{D_{1}}}\sum_{i\in D_{1}\cap s}\left[(Y_{i,t+1}^{1}-Y_{i,t}^{0})-\sum_{j\in D_{0}\cap s}w_{ij}\left(Y_{j,t+1}^{0}-Y_{j,t}^{0}\right)\right],$$ (1)

where D_1_(D_0_) represents the intervention(control) group, t(t+1) denotes the pre-(post-) intervention period, w_ij_ identifies the NN matching weight, and S stands for the area of common support.

The model above was estimated separately for each outcome to obtain the insurance effects. Standard errors were obtained by bootstrapping, and heterogeneity analysis was also performed by splitting the sample into subsamples. All PSM analyses were performed using Stata ado psmatch2 [37].

## Results

### Descriptive analysis

Table 1 describes the characteristics and outcomes of the control and intervention groups in the two waves. In the 2006 wave, age was concentrated in the 40–60 range in the control group and >60 in the intervention group. Compared to participants in the control group, those in the intervention group were significantly less likely to be married, well educated, or employed with a high income but were more likely to have more family members (all Ps<0.01). However, the comparison of self-reported health and chronic disease presented no statistical difference on the baseline. In terms of behaviors, from 2006 to 2011, the probability of preventive care service utilization decreased in the intervention group compared to an increase in the control group, showing a significant difference in the 2011 wave. Meanwhile, individuals in the intervention group tended to increase their probability of drinking soft drinks and engaging in physical activity more than the control group after the insurance reform (P<0.05).

**Table 1:** Characteristics of participants [%(95% CIs)].

Variables	Control		Intervention		P-value for difference	P-value for difference
	(n = 378)		(n = 225)			
	Before reform	After reform	Before reform	After reform	(2006)	(2011)
Panel 1: Preventive care service utilization						
Preventive care service utilization						
No	90.74	90.45	95.11	96.43	0.051	0.007
	(87.36–93.47)	(87.03–93.22)	(91.42–97.53)	(93.08–98.45)		
Yes	9.26	9.55	4.89	3.57		
	(6.53–12.64)	(6.78–12.97)	(2.47–8.58)	(1.55–6.92)		
Panel 2: Health behaviors						
Smoke						
No	74.34	75.93	80.89	78.67	0.065	0.440
	(69.62–78.67)	(71.29–80.15)	(75.13–85.81)	(72.73–83.83)		
Yes	25.66	24.07	19.11	21.33		
	(21.33–30.38)	(19.85–28.71)	(14.19–24.87)	(16.17–27.27)		
Drink						
No	62.96	64.02	68.44	69.78	0.172	0.148
	(57.88–67.85)	(58.96–68.87)	(61.94–74.46)	(63.32–75.70)		
Yes	37.04	35.98	31.56	30.22		
	(32.15–42.12)	(31.13–41.04)	(25.54–38.06)	(24.30–36.68)		
Soft drink						
No	76.13	70.63	73.21	62.22	0.425	0.033
	(71.50–80.34)	(65.76–75.18)	(66.91–78.89)	(55.54–68.58)		
Yes	23.87	29.37	26.79	37.78		
	(19.66–28.50)	(24.82–34.24)	(21.11–33.09)	(31.42–44.46)		
Physical activity						
No	71.69	68.97	83.56	79.56	0.001	0.005
	(66.86–76.18)	(64.03–73.60)	(78.05–88.15)	(73.69–84.63)		
Yes	28.31	31.03	16.44	20.44		
	(23.82–33.14)	(26.40–35.97)	(11.85–21.95)	(15.37–26.31)		
Sedentary						
No	0.26	2.38	4.05	1.33	0.0001	0.373
	(0.01–1.47)	(1.09–4.47)	(1.87–7.56)	(0.27–3.85)		
Yes	99.74	97.62	95.95	98.67		
	(98.53–99.99)	(95.53–98.91)	(92.44–98.13)	(96.15–99.72)		
Overweight						
No	67.13	64.61	63.98	62.16	0.443	0.548
	(62.01–71.97)	(59.52–69.47)	(57.11–70.46)	(55.43–68.57)		
Yes	32.87	35.39	36.02	37.84		
	(28.03–37.99)	(30.54–40.48)	(29.54–42.89)	(31.43–44.57)		
Panel 3: Demographic and socioeconomic characteristics						
Age (years)						
18–45	28.31	15.87	34.22	23.56	0.001	0.016
	(23.82–33.14)	(12.34–19.95)	(28.05–40.82)	(18.17–29.65)		
46–60	46.03	41.01	30.67	31.11		
	(40.92–51.20)	(36.00–46.15)	(24.71–37.14)	(25.13–37.60)		
>60	25.66	43.12	35.11	45.33		
	(21.33–30.38)	(38.07–48.28)	(28.89–41.73)	(38.71–52.09)		
Gender						
Male	50.79	50.79	40.00	40.00	0.010	0.010
	(45.63–55.94)	(45.63–55.94)	(33.55–46.72)	(33.55–46.72)		
Female	49.21	49.21	60.00	60.00		
	(44.06–54.37)	(44.06–54.37)	(53.28–66.45)	(53.28–66.45)		
Marriage						
Others	9.52	13.03	19.11	21.52	0.001	0.006
	(6.76–12.94)	(9.80–16.86)	(14.19–24.87)	(16.32–27.51)		
Married	90.48	86.97	80.89	78.48		
	(87.06–93.24)	(83.14–90.20)	(75.13–85.81)	(72.49–83.68)		
Educational level						
Primary school and below	15.87	15.12	48.00	47.32	0.0001	0.0001
	(12.34–19.95)	(11.66–19.14)	(41.31–54.74)	(40.63–54.08)		
Junior or senior high school	39.15	37.93	47.11	45.98		
	(34.20–44.28)	(33.01–43.04)	(40.44–53.86)	(39.32–52.75)		
College and above	44.98	46.95	4.89	6.70		
	(39.88–50.14)	(41.82–52.13)	(2.47–8.58)	(3.80–10.80)		
Job						
No	44.71	58.47	75.56	73.78	0.0001	0.0001
	(39.62–49.88)	(53.32–63.48)	(69.40–81.02)	(67.52–79.40)		
Yes	55.29	41.53	24.44	26.22		
	(50.12–60.38)	(36.52–46.68)	(18.98–30.60)	(20.60–32.48)		
Household income						
Low	15.61	5.03	54.22	34.67	0.0001	0.0001
	(12.10–19.67)	(3.05–7.74)	(47.47–60.86)	(28.47–41.28)		
Middle	57.41	29.37	30.67	32.44		
	(52.25–62.45)	(24.82–34.24)	(24.71–37.14)	(26.38–38.99)		
High	26.98	65.60	15.11	32.89		
	(22.58–31.76)	(60.58–70.39)	(10.70–20.47)	(26.79–39.45)		
Household size						
≤2	43.12	43.12	32.44	32.44	0.003	0.003
	(38.07–48.28)	(38.07–48.28)	(26.37–38.99)	(26.37–38.99)		
= 3	31.22	31.22	27.56	27.56		
	(26.58–36.15)	(26.58–36.15)	(21.83–33.89)	(21.83–33.89)		
= 4	15.34	15.34	24.00	24.00		
	(11.86–19.38)	(11.86–19.38)	(18.57–30.13)	(18.57–30.13)		
≥5	10.32	10.32	16.00	16.00		
	(7.44–13.83)	(7.44–13.83)	(11.46–21.46)	(11.46–21.46)		
Region						
East area	35.98	35.98	31.56	31.56	0.520	0.520
	(31.13–41.04)	(31.13–41.04)	(25.54–38.06)	(25.54–38.06)		
Middle area	52.91	52.91	57.33	57.33		
	(47.74–58.03)	(47.74–58.03)	(50.59–63.88)	(50.59–63.88)		
West area	11.11	11.11	11.11	11.11		
	(8.13–14.72)	(8.13–14.72)	(7.32–15.96)	(7.32–15.96)		
Panel 4: Health status						
Self-reported health						
Poor	41.53	-	38.67	-	0.488	-
	(36.52–46.68)		(32.27–45.37)			
Good	58.47	-	61.33	-		
	(53.32–63.48)		(54.63–67.73)			
Chronic disease						
No	83.33	72.34	81.78	72.00	0.625	0.928
	(79.19–86.95)	(67.52–76.80)	(76.10–86.60)	(65.65–77.76)		
Yes	16.67	27.66	18.22	28.00		
	(13.05–20.81)	(23.20–32.48)	(13.41–23.90)	(22.24–34.35)		

### Influencing factors for residents participating in URBMI

Table 2 presents the probit estimates for the influencing factors among residents participating in URBMI. Among all sociodemographic characteristics, age, education, job status, and income were all significantly associated with being URBMI insured. As the table shows, there was a negative correlation between age and insurance, implying that younger residents were more likely to participate in URBMI (P<0.01). Compared to the control individuals, those with a higher educational level were less likely to participate in URBMI (P<0.001). In addition, urban residents without a job or with lower incomes were more inclined to participate in URBMI (P<0.001).

**Table 2:** Probit estimates of the probability of being URBMI insured.

Variables	β	S.E.	Z	P
Age 46–60	-0.821	0.176	-4.66	<0.0001
Age>60	-0.689	0.23	-2.99	0.003
Female	-0.055	0.133	-0.42	0.678
Married	-0.165	0.18	-0.92	0.359
Junior or senior high school	-0.05	0.162	-3.1	0.002
College and above	-1.821	0.226	-8.06	<0.0001
Have a job	-0.838	0.165	-5.07	<0.0001
Middle income	-0.801	0.144	-5.57	<0.0001
High income	-0.64	0.183	-3.5	<0.0001
Household size = 3	0.274	0.169	1.62	0.105
Household size = 4	0.395	0.184	2.15	0.032
Household size≥5	0.359	0.2	1.79	0.073
Middle area	0.012	0.139	0.09	0.929
West area	0.305	0.24	1.27	0.203
Self-report good	0.249	0.134	1.85	0.064
Have chronic	-0.08	0.178	-0.45	0.651
Constant	1.437	0.334	4.31	<0.0001
LR χ^2^	259.78			
P>χ^2^	<0.0001			
Pseudo R^2^	0.3261			
Observations	603			

### Effect of insurance on preventive activities and health behaviors

After restricting the analyses to individuals in the common support range (0.017, 0.995), matching analyses were imposed restrictively on 550 respondents. Table 3 reports the main estimates of the effect of URBMI on preventive care service utilization and health behavior. For intervened individuals, the probability of sedentariness increased significantly by 5.1%, which is in line with the results for unmatched individuals. Being URBMI insured increased the likelihood of smoking by 1.2%, but the result was not statistically significant. The probability of using preventive care services, drinking alcohol, consuming soft drinks, performing physical activity, and being overweight did not change significantly after urban residents obtained URBMI.

**Table 3:** Average treatment effects on the intervention (ATT).

Variables	Unmatched		Matched		
	DIFF	SE	ATT	SE	N_t_/N_c_
Preventive care service utilization	0.026	0.031^a^	0.040	0.067	214/324^b^
Smoking	0.05	0.053	0.012	0.107	214/325
Drinking	0.067	0.056	-0.004	0.096	214/325
Soft drinks	-0.006	0.057	-0.060	0.089	213/324
Physical activity	0.001	0.049	-0.070	0.096	214/325
Sedentariness	0.061***^c^	0.015	0.051***	0.023	211/325
Obesity	-0.004	0.060	0.002	0.108	200/303

^a^Bootstrapped standard errors of matched individuals are reported (200 repetitions).

^b^N_t_ = number in intervention group; N_c_ = number in control group.

^c^*, **, and *** denote statistically significant differences at the 10%, 5%, and 1% levels, respectively.

### Effect of insurance on different subgroups

Table 4 presents the results for average treatment effects among different subgroups. Similar to the above analyses, the probability of sedentariness increased among those reporting both poor and good health (P<0.10). Meanwhile, residents with poor self-reported health reduced the probability of exercise by 27.5% (P<0.05). Females tended to be more sedentary (P<0.05), as did low- and middle-income individuals (P<0.10). With respect to soft drinks, male enrollees tended to be less likely to drink soft drinks by 22.6% (P<0.10). Regarding the probability of smoking, middle-income enrollees tended to be more likely to smoke (P<0.10), but no significant results were observed for the poor or rich.

**Table 4:** Average treatment effects in different subgroups.

Variables	By gender		By self-reported health		By household income (per capita)		
	Male	Female	Poor	Good	Low	Middle	High
Preventive care service utilization	-0.118	0.125	0.063	0.023	-0.030	0.048	0.209
	(0.074)^a^	(0.089)	(0.110)	(0.077)	(0.108)	(0.077)	(0.144)
Smoking	0.212	-0.070	-0.038	0.083	0.000	0.226*	-0.047
	(0.148)	(0.102)	(0.144)	(0.130)	(0.159)	(0.133)	(0.161)
Drinking	-0.082	0.125	-0.038	0.060	0.109	0.065	0.000
	(0.136)	(0.107)	(0.135)	(0.125)	(0.159)	(0.148)	(0.189)
Soft drinks	-0.226*^b^	-0.016	-0.310	-0.038	0.170	-0.210	-0.279
	(0.129)	(0.128)	(0.141)	(0.114)	(0.132)	(0.139)	(0.256)
Physical activity	-0.118	0.023	-0.275**	0.000	-0.119	-0.065	-0.209
	(0.120)	(0.112)	(0.109)	(0.108)	(0.144)	(0.085)	(0.203)
Sedentariness	0.048	0.071**	0.100*	0.038*	0.080*	0.049*	-0.024
	(0.030)	(0.029)	(0.058)	(0.021)	(0.046)	(0.028)	(0.058)
Obesity	0.063	-0.025	0.107	-0.096	0.258	-0.169	0.275
	(0.145)	(0.118)	(0.142)	(0.134)	(0.164)	(0.167)	(0.253)

^a^Bootstrapped standard errors are reported (200 repetitions).

^b^*, **, and *** denote statistically significant differences at the 10%, 5%, and 1% levels, respectively.

## Discussion

This study aimed to reveal the effects of China’s urban basic health insurance on individuals’ behaviors. Using the CHNS and combining two approaches—difference-in-differences and propensity score matching—we assessed the effects of a basic insurance scheme through comparison with a previously uninsured group. The sample was composed of adults over 18 years of age. In addition, owing to the implementation of URBMI in 2007, we were able to focus on the completely uninsured group rather than beneficiaries of preexisting insurance schemes and could then distinguish between the uninsured group and those enrolled in insurance schemes (mainly UEBMI) [24].

The empirical results indicated that URBMI did not significantly change preventive care utilization. This does not support the findings of Baicker, who found a positive effect of Medicaid on the utilization of preventive services such as cholesterol screening, mammography, and prostate cancer screening in the Oregon Health Experiment [38]. Although such preventive measures are financed by the insurance scheme—which may incentivize prevention as it makes preventive care less costly [12, 39]—some studies have found that the demand for preventive care may be relatively inelastic, perhaps due to long waiting times or uncomfortable experiences [40–42]. URBMI is a government-run voluntary insurance program targeting the prevention of serious illnesses by providing a service package for basic health and against catastrophic diseases. The effects of URBMI on preventive care use are probably negligible since the health insurance offers incomplete coverage [43].

Regarding the effects on unhealthy or risky behaviors, our analyses found no evidence that health insurance coverage increased soft drink consumption, physical activity, or obesity between the focus and control groups. Our results suggested a small but measurable problem that URBMI participation, on average, increased the probability of tobacco use by 1.2%. Although this result is not statistically significant, it should be a cause for concern since smoking is universally recognized as an unhealthy behavior. Our results also suggested an approximately 5% increase in the probability of sedentariness after participating in URBMI, which aligns with prior studies [11–13]. Consistent with the literature, little association was found between sedentary behavior and physical activity in our study, most likely because of the different measurements [44–46]. Our definition and operationalization of physical activity is consistent with most previous studies, comprising moderate to vigorous physical activity, while sedentary behavior was measured in accordance with the suggestion of the “Sedentary Behavior Research Network,” where sedentariness is distinguished from “inactivity” and describes waking behavior characterized by low-energy expenditure in a sitting or reclining posture [47]. Overall, the effect of health insurance on health behaviors is ambiguous. On the one hand, health insurance does not directly insure against health risks but only the financial consequences of illness; thus, it might not sufficiently incentivize engagement in healthy behaviors, which in turn incurs negative effects on health with cross-price effects [14, 17]. On the other hand, the insurance only reimburses a portion of treatment costs, and physical pain and opportunity cost continue to exist in the process of treatment and recovery. Meanwhile, health insurance can increase the likelihood of contact with physicians, who may advise patients to adopt healthy behaviors, which is likely to positively influence the health behaviors of insured persons [20, 48, 49].

Our main models included all adults, but we also estimated models separately by gender, self-reported health, and income because behavioral responses may vary by subpopulation. Prior studies have suggested that men and women may respond differently to insurance coverage [50]. According to our results, an increase of 7.1% for sedentariness was seen in the female group, which is in line with Qin’s results [7]. Among males, URBMI was associated with a negative effect on drinking soft drinks. In addition, residents with self-reported poor health tended to exercise less while those who reported good health were more likely to be sedentary. The results from our subgroup analysis by household income status are consistent with the theory that decreased work-related income can have a negative effect on health [51]. Our results suggest an elevated frequency of sedentariness among individuals who are not rich. Middle-income enrollees tended to be more likely to smoke, but no significant results were found for the poor or rich.

An important issue that should be noted in the interpretation of the results is that lost panel data could be a source of bias if the loss of follow-up was not random [52]. To address this issue, we compared the characteristics of follow-up status in the 2006 base wave. The results are presented in S1 Table. No significant differences were found in the outcomes—except for soft drink consumption—between follow-up individuals and those who were not followed up. Another issue pertains to the study’s methodology. Our analysis was based on panel data rather than cross-sectional data, which differs from prior studies but allows for the control of unobservable time-invariant factors. The combination of PSM and DID provides more robust results for estimating treatment effect due to removing biases caused by covariance [35]. The matching results are presented in S2 Table. Before the match, there were significant differences between the intervention and control groups in terms of gender, marriage, educational level, job status, and household income. After the propensity score matching, the differences between all variables in the intervention and control groups were no longer statistically significant. The results indicate that the PSM method can reduce the difference in observed characteristics before insurance participation. Restrictions on the range of common support substantially reduce differences in the observed variables and control for residual differences. Additionally, the DID model relaxes the PSM restrictions, making model-based adjustments less sensitive to other unobservable variables. This reduced sensitivity again facilitates the estimation of parametric approximations of ATT [53].

The policy implication of our findings is that measures should be adopted to encourage preventive activities and healthy behaviors. For this purpose, a health insurance benefit package that attaches preventive health care programs could be effective. Specifically, this could involve financial incentives such as removing cost sharing for preventive care and providing cash rewards or penalties, respectively, for decreases or increases in unhealthy behavior. Another possible alternative could be built on the peer effect. One study found that employees in the same company tended to join the same insurance schemes, implying that insurance companies may contribute to health choices by promoting healthy lunch menus or other related options [13]. Lastly, increasing access to doctors and reducing barriers to information could play a role in promoting healthy lifestyles. Not only are doctors expected to advise their patients in precautionary behaviors, but health promotion and education can also be used to promote healthy lifestyles.

This study has a number of limitations. First, the results have limited generalizability given the restrictions we placed on our sample. In particular, the restrictions we imposed on the types of health insurance led to a limited sample, which was mainly affected by the integration process in medical insurance reform during the survey period. Regarding the data, it should be noted that incomplete data resulted in follow-up loss, and objective measurements of health behaviors could have led to misreporting. Lastly, the long-term effects of URBMI remain unknown due to unavailable data. Despite these limitations, this study provides some empirical evidence regarding the effects of China’s urban basic health insurance on preventive care service utilization and changes in health behavior.

## Conclusions

Using the data drawn from the CHNS during the period 2006–2011, we found that the utilization of preventive care did not change significantly after the URBMI reform. We also found that while sedentariness increased among urban residents, other unhealthy behaviors such as smoking and drinking did not, nor were there increases in obesity. In addition, different subsamples reacted differently. Female and low- or middle-income enrollees were more likely to be sedentary, people with poor health tended to exercise less, and middle-income enrollees were more likely to smoke. It is essential to increase awareness regarding the importance of preventive activities and healthy behaviors, especially among newly insured individuals.

## Supporting information

S1 Table Differences between follow-up and loss of follow-up samples. (DOCX)

S2 Table Covariate balance results after propensity score matching. (DOCX)
