# Supplementary material for: Effects of China’s urban basic health insurance on preventive care service utilization and health behaviors: Evidence from the China Health and Nutrition Survey
Source: PLoS One. 2018 Dec 31;13(12):e0209890. doi: 10.1371/journal.pone.0209890 (PMC6312240; doi:10.1371/journal.pone.0209890)
Supplement: S1 Table — (DOCX) [file pone.0209890.s001.docx]

**S1 Table. Differences between the** **follow-up and** **loss of follow-up samples[**$\boldsymbol{\%(95\%CI)}$**]**

| **Variables** | **Follow-up**  **(n=1,934)** | **Lose to follow-up (n=1,268)** | **P-value for difference** |
| --- | --- | --- | --- |
| Preventive care service utilization |  |  |  |
| No | 94.83(93.75-95.77) | 95.27(93.95-96.37) | 0.577 |
| Yes | 5.17(4.23-6.25) | 4.73(3.63-6.05) |  |
| Smoke |  |  |  |
| No | 72.54(70.50-74.52) | 74.01(71.50-76.41) | 0.359 |
| Yes | 27.46(25.48-29.50) | 25.99(23.59-28.50) |  |
| Drink |  |  |  |
| No | 65.72(63.55-67.83) | 66.93(64.26-69.52) | 0.479 |
| Yes | 34.28(32.17-36.45) | 33.07(30.48-35.74) |  |
| Soft drink |  |  |  |
| No | 73.89(71.87-75.83) | 68.48(65.84-71.04) | 0.001 |
| Yes | 26.11(24.17-28.13) | 31.52(28.96-34.16) |  |
| Physical activity |  |  |  |
| No | 81.02(79.19-82.75) | 78.76(76.40-81.00) | 0.119 |
| Yes | 18.98(17.25-20.81) | 21.24(19.01-23.60) |  |
| Sedentary |  |  |  |
| No | 3.89(3.08-4.86) | 5.37(4.19-6.76) | 0.048 |
| Yes | 96.11(95.14-96.92) | 94.63(93.24-95.81) |  |
| Overweight |  |  |  |
| No | 68.99(66.81-71.11) | 72.30(69.59-74.89) | 0.056 |
| Yes | 31.01(28.89-33.19) | 27.70(25.11-30.41) |  |
| Age(years) |  |  |  |
| 18-45 | 38.00(35.83-40.21) | 44.94(42.17-47.73) | <0.0001 |
| 46-60 | 36.50(34.35-38.70) | 26.82(24.39-29.35) |  |
| >60 | 25.50(23.56-27.50) | 28.24(25.77-30.81) |  |
| Gender |  |  |  |
| Male | 47.26(45.01-49.51) | 48.58(45.80-51.37) | 0.464 |
| Female | 52.74(50.49-54.99) | 51.42(48.63-54.20) |  |
| Marriage |  |  |  |
| Others | 14.27(12.74-15.91) | 25.63(23.25-28.13) | <0.0001 |
| Married | 85.73(84.09-87.26) | 74.37(71.87-76.75) |  |
| Educational level |  |  |  |
| Primary school and below | 34.07(31.96-36.24) | 29.10(26.61-31.69) | <0.0001 |
| Junior or senior high school | 46.43(44.19-48.68) | 43.45(40.70-46.23) |  |
| College and above | 19.50(17.75-21.33) | 27.45(25.00-29.99) |  |
| Job |  |  |  |
| No | 52.59(50.33-54.83) | 51.26(48.47-54.05) | 0.463 |
| Yes | 47.41(45.17-49.67) | 48.74(45.95-51.53) |  |
| Household income(Ren Min Bi) |  |  |  |
| Low | 44.31(42.08-46.56) | 43.77(41.02-46.55) | 0.482 |
| Middle | 40.85(38.65-43.08) | 39.83(37.12-42.58) |  |
| High | 14.84(13.28-16.50) | 16.40(14.41-18.56) |  |
| Household size |  |  |  |
| ≤2 | 28.39(26.39-30.45) | 31.23(28.69-33.86) | 0.291 |
| = 3 | 31.18(29.12-33.30) | 29.42(26.92-32.01) |  |
| = 4 | 21.51(19.70-23.41) | 20.03(17.86-22.34) |  |
| ≥5 | 18.92(17.20-20.74) | 19.32(17.18-21.60) |  |
| Region |  |  | <0.0001 |
| East area | 30.82(28.76-32.93) | 39.35(36.65-42.10) |  |
| Middle area | 48.14(45.89-50.39) | 40.70(37.97-43.46) |  |
| West area | 21.04(19.25-22.93) | 19.95(17.79-22.26) |  |
| Self-report health |  |  |  |
| Poor | 42.24(40.03-44.48) | 44.16(41.41-46.95) | 0.283 |
| Good | 57.76(55.52-59.97) | 55.84(53.05-58.59) |  |
| Chronic disease |  |  |  |
| No | 84.69(83.01-86.27) | 81.78(79.55-83.87) | 0.030 |
| Yes | 15.31(13.73-16.99) | 18.22(16.13-20.45) |  |
