# Supplementary material for: Effects of China’s urban basic health insurance on preventive care service utilization and health behaviors: Evidence from the China Health and Nutrition Survey
Source: PLoS One. 2018 Dec 31;13(12):e0209890. doi: 10.1371/journal.pone.0209890 (PMC6312240; doi:10.1371/journal.pone.0209890)
Supplement: S2 Table — (DOCX) [file pone.0209890.s002.docx]

**S2 Table. Covariate balance results after propensity score matching**

| **Variable** | **Unmatched** | **Outcomes** | | | | | | | | | | | | | | |  |
| --- | --- | --- | --- | --- | --- | --- | --- | --- | --- | --- | --- | --- | --- | --- | --- | --- | --- |
|  |  | **Preventive care services utilization** | | |  | **Smoke** | | |  | **Drink** | | |  | **Soft drink** | | | |
|  | **Matched** | **Mean** | | **t** |  | **Mean** | | **t** |  | **Mean** | | **t** |  | **Mean** | | **t** | |
|  |  | **Intervention** | **Control** |  |  | **Intervention** | **Control** |  |  | **Intervention** | **Control** |  |  | **Intervention** | **Control** |  | |
| Age 46-60 | U | 0.31 | 0.38 | -1.66* |  | 0.31 | 0.38 | -1.70* |  | 0.31 | 0.38 | -1.70* |  | 0.31 | 0.38 | -1.67* | |
|  | M | 0.31 | 0.34 | -0.58 |  | 0.31 | 0.34 | -0.58 |  | 0.31 | 0.34 | -0.58 |  | 0.31 | 0.34 | -0.52 | |
| Age >60 | U | 0.35 | 0.29 | 1.42 |  | 0.35 | 0.29 | 1.45 |  | 0.35 | 0.29 | 1.45 |  | 0.35 | 0.29 | 1.38 | |
|  | M | 0.35 | 0.37 | -0.47 |  | 0.35 | 0.37 | -0.47 |  | 0.35 | 0.37 | -0.47 |  | 0.35 | 0.37 | -0.53 | |
| Female | U | 0.60 | 0.51 | 2.03** |  | 0.60 | 0.51 | 2.07** |  | 0.60 | 0.51 | 2.07** |  | 0.60 | 0.51 | 2.09** | |
|  | M | 0.60 | 0.60 | -0.001 |  | 0.60 | 0.60 | -0.001 |  | 0.60 | 0.60 | -0.001 |  | 0.61 | 0.60 | 0.04 | |
| Married | U | 0.81 | 0.90 | -3.12*** |  | 0.81 | 0.90 | -3.01*** |  | 0.81 | 0.90 | -3.01*** |  | 0.81 | 0.90 | -2.88*** | |
|  | M | 0.80 | 0.80 | 0.07 |  | 0.80 | 0.80 | 0.10 |  | 0.80 | 0.80 | 0.10 |  | 0.81 | 0.80 | 0.22 | |
| Junior or senior high school | U | 0.47 | 0.46 | 0.33 |  | 0.47 | 0.46 | 0.36 |  | 0.47 | 0.46 | 0.36 |  | 0.47 | 0.46 | 0.38 | |
|  | M | 0.48 | 0.45 | 0.48 |  | 0.48 | 0.45 | 0.48 |  | 0.48 | 0.45 | 0.48 |  | 0.48 | 0.45 | 0.52 | |
| College and above | U | 0.05 | 0.36 | -9.04*** |  | 0.05 | 0.36 | -9.09*** |  | 0.05 | 0.36 | -9.09*** |  | 0.05 | 0.36 | -9.01*** | |
|  | M | 0.05 | 0.05 | -0.22 |  | 0.05 | 0.05 | -0.22 |  | 0.05 | 0.05 | -0.22 |  | 0.05 | 0.05 | -0.22 | |
| Have a job | U | 0.24 | 0.50 | -6.22*** |  | 0.24 | 0.50 | -6.26*** |  | 0.24 | 0.50 | -6.26*** |  | 0.25 | 0.50 | -6.18*** | |
|  | M | 0.26 | 0.25 | 0.09 |  | 0.26 | 0.25 | 0.07 |  | 0.26 | 0.25 | 0.07 |  | 0.26 | 0.26 | 0.07 | |
| Income middle | U | 0.31 | 0.56 | -6.13*** |  | 0.31 | 0.56 | -6.17*** |  | 0.31 | 0.57 | -6.17*** |  | 0.31 | 0.57 | -6.13*** | |
|  | M | 0.31 | 0.31 | -0.07 |  | 0.31 | 0.31 | -0.27 |  | 0.31 | 0.31 | -0.07 |  | 0.31 | 0.31 | -0.07 | |
| Income high | U | 0.15 | 0.25 | -24.70*** |  | 0.15 | 0.25 | -2.77*** |  | 0.15 | 0.25 | -2.77*** |  | 0.15 | 0.25 | -2.74*** | |
|  | M | 0.15 | 0.14 | -0.60 |  | 0.15 | 0.14 | 0.27 |  | 0.15 | 0.14 | 0.27 |  | 0.15 | 0.14 | 0.27 | |
| Household size=3 | U | 0.28 | 0.32 | -1.14 |  | 0.28 | 0.32 | -1.19 |  | 0.28 | 0.32 | -1.19 |  | 0.28 | 0.32 | -1.11 | |
|  | M | 0.27 | 0.28 | -0.11 |  | 0.27 | 0.28 | -0.13 |  | 0.27 | 0.28 | -0.13 |  | 0.27 | 0.28 | -0.13 | |
| Household size=4 | U | 0.24 | 0.18 | 1.84* |  | 0.24 | 0.18 | 1.86 |  | 0.24 | 0.18 | 1.86* |  | 0.24 | 0.18 | 1.87* | |
|  | M | 0.24 | 0.21 | 0.60 |  | 0.24 | 0.21 | 0.62 |  | 0.24 | 0.21 | 0.62 |  | 0.24 | 0.21 | 0.62 | |
| Household size>=5 | U | 0.16 | 0.12 | 1.33 |  | 0.16 | 0.12 | 1.34 |  | 0.16 | 0.12 | 1.32 |  | 0.16 | 0.12 | 1.35 | |
|  | M | 0.16 | 0.19 | -0.82 |  | 0.16 | 0.19 | -0.82 |  | 0.16 | 0.19 | -0.82 |  | 0.16 | 0.19 | -0.72 | |
| Middle area | U | 0.57 | 0.52 | 1.12 |  | 0.57 | 0.53 | 1.29 |  | 0.57 | 0.53 | 1.09 |  | 0.57 | 0.53 | 1.01 | |
|  | M | 0.58 | 0.57 | 0.29 |  | 0.58 | 0.57 | 0.29 |  | 0.58 | 0.57 | 0.29 |  | 0.58 | 0.56 | 0.27 | |
| West area | U | 0.11 | 0.10 | 0.35 |  | 0.11 | 0.10 | 0.36 |  | 0.11 | 0.10 | 0.36 |  | 0.11 | 0.10 | 0.48 | |
|  | M | 0.09 | 0.08 | 0.66 |  | 0.09 | 0.08 | 0.66 |  | 0.09 | 0.08 | 0.66 |  | 0.09 | 0.08 | 0.66 | |
| Self-report good | U | 0.61 | 0.60 | 0.42 |  | 0.61 | 0.60 | 0.39 |  | 0.61 | 0.60 | 0.39 |  | 0.61 | 0.60 | 0.37 | |
|  | M | 0.62 | 0.64 | -0.50 |  | 0.62 | 0.64 | -0.50 |  | 0.62 | 0.64 | -0.50 |  | 0.62 | 0.65 | -0.54 | |
| Have chronic | U | 0.18 | 0.17 | 0.28 |  | 0.18 | 0.17 | 0.30 |  | 0.18 | 0.17 | 0.30 |  | 0.18 | 0.17 | 0.31 | |
|  | M | 0.19 | 0.20 | -0.37 |  | 0.19 | 0.20 | -0.34 |  | 0.19 | 0.20 | -0.34 |  | 0.19 | 0.20 | -0.25 | |
| LR χ2 | U | 190.76*** | | |  | 191.50*** | | |  | 191.50*** | | |  | 189.49*** | | | |
|  | M | 2.28 | | |  | 2.31 | | |  | 2.31 | | |  | 2.28 | | | |

**S2 Table. Covariate balance results after propensity score matching (Continued)**

| **Variable** | **Unmatched** | **Outcomes** | | | | | | | | | | |
| --- | --- | --- | --- | --- | --- | --- | --- | --- | --- | --- | --- | --- |
|  |  | **Activity** | | |  | **Sedentary** | | |  | **Overweight** | | |
|  | **Matched** | **Mean** | | **t** |  | **Mean** | | **t** |  | **Mean** | | **t** |
|  |  | **Intervention** | **Control** |  |  | **Intervention** | **Control** |  |  | **Intervention** | **Control** |  |
| Age 46-60 | U | 0.31 | 0.38 | -1.70* |  | 0.31 | 0.38 | -1.71* |  | 0.31 | 0.38 | -1.61 |
|  | M | 0.31 | 0.34 | -0.58 |  | 0.31 | 0.34 | -0.56 |  | 0.32 | 0.33 | -0.24 |
| Age >60 | U | 0.35 | 0.29 | 1.45 |  | 0.35 | 0.29 | 1.34 |  | 0.37 | 0.31 | 1.43 |
|  | M | 0.35 | 0.37 | -0.47 |  | 0.35 | 0.37 | -0.53 |  | 0.37 | 0.39 | -0.48 |
| Female | U | 0.60 | 0.51 | 2.07* |  | 0.60 | 0.51 | 2.04** |  | 0.60 | 0.52 | 1.69* |
|  | M | 0.60 | 0.60 | -0.001 |  | 0.60 | 0.60 | -0.06 |  | 0.61 | 0.61 | -0.16 |
| Married | U | 0.81 | 0.90 | -3.01*** |  | 0.81 | 0.90 | -2.94*** |  | 0.82 | 0.90 | -2.56** |
|  | M | 0.80 | 0.80 | 0.10 |  | 0.81 | 0.80 | 0.22 |  | 0.82 | 0.81 | 0.38 |
| Junior or senior high school | U | 0.47 | 0.46 | 0.36 |  | 0.48 | 0.46 | 0.51 |  | 0.47 | 0.46 | 0.18 |
|  | M | 0.48 | 0.45 | 0.48 |  | 0.48 | 0.45 | 0.62 |  | 0.47 | 0.44 | 0.58 |
| College and above | U | 0.05 | 0.36 | -9.09*** |  | 0.05 | 0.36 | -9.01*** |  | 0.04 | 0.35 | -8.70*** |
|  | M | 0.05 | 0.05 | -0.22 |  | 0.05 | 0.05 | -0.22 |  | 0.04 | 0.05 | -0.66 |
| Have a job | U | 0.24 | 0.50 | -6.26*** |  | 0.25 | 0.50 | -6.14*** |  | 0.24 | 0.49 | -5.91*** |
|  | M | 0.26 | 0.25 | 0.07 |  | 0.26 | 0.26 | 0.11 |  | 0.25 | 0.24 | 0.33 |
| Income middle | U | 0.31 | 0.57 | -6.17*** |  | 0.31 | 0.57 | -6.15*** |  | 0.33 | 0.57 | -5.67*** |
|  | M | 0.31 | 0.31 | -0.07 |  | 0.31 | 0.31 | -0.05 |  | 0.33 | 0.32 | -0.03 |
| Income high | U | 0.15 | 0.25 | -2.77*** |  | 0.15 | 0.25 | -2.83*** |  | 0.15 | 0.24 | -2.24** |
|  | M | 0.15 | 0.14 | 0.27 |  | 0.15 | 0.15 | 0.14 |  | 0.16 | 0.14 | 0.50 |
| Household size=3 | U | 0.28 | 0.32 | -1.19 |  | 0.27 | 0.32 | -1.32 |  | 0.27 | 0.32 | -1.10 |
|  | M | 0.27 | 0.28 | -0.13 |  | 0.27 | 0.28 | -0.33 |  | 0.27 | 0.26 | 0.02 |
| Household size=4 | U | 0.24 | 0.18 | 1.86 |  | 0.24 | 0.18 | 1.94* |  | 0.23 | 0.17 | 1.73* |
|  | M | 0.24 | 0.21 | 0.62 |  | 0.24 | 0.22 | 0.65 |  | 0.23 | 0.22 | 0.29 |
| Household size>=5 | U | 0.16 | 0.12 | 1.34 |  | 0.16 | 0.12 | 1.41 |  | 0.17 | 0.12 | 1.66* |
|  | M | 0.16 | 0.19 | -0.82 |  | 0.16 | 0.19 | -0.67 |  | 0.17 | 0.18 | -0.34 |
| Middle area | U | 0.57 | 0.53 | 1.09 |  | 0.57 | 0.53 | 0.95 |  | 0.58 | 0.52 | 1.22 |
|  | M | 0.58 | 0.57 | 0.29 |  | 0.57 | 0.56 | 0.20 |  | 0.58 | 0.57 | 0.20 |
| West area | U | 0.11 | 0.10 | 0.36 |  | 0.11 | 0.10 | 0.41 |  | 0.11 | 0.11 | 0.14 |
|  | M | 0.09 | 0.08 | 0.66 |  | 0.09 | 0.08 | 0.66 |  | 0.10 | 0.07 | 0.83 |
| Self-report good | U | 0.61 | 0.60 | 0.39 |  | 0.61 | 0.60 | 0.26 |  | 0.62 | 0.59 | 0.57 |
|  | M | 0.62 | 0.64 | -0.50 |  | 0.62 | 0.64 | -0.60 |  | 0.63 | 0.62 | 0.04 |
| Have chronic | U | 0.18 | 0.17 | 0.30 |  | 0.18 | 0.17 | 0.24 |  | 0.19 | 0.18 | 0.03 |
|  | M | 0.19 | 0.20 | -0.34 |  | 0.18 | 0.20 | -0.37 |  | 0.19 | 0.21 | -0.57 |
| LR χ2 | U | 191.50*** | | |  | 189.31*** | | |  | 170.46*** | | |
|  | M | 2.31 | | |  | 2.78 | | |  | 2.17 | | |
